# Effects of combined MAO-B inhibitors and levodopa vs. monotherapy in Parkinson’s disease

**DOI:** 10.3389/fnagi.2014.00180

**Published:** 2014-07-25

**Authors:** Rakhee Krishna, Manal Ali, Ahmed A. Moustafa

**Affiliations:** ^1^Department of Psychiatry, Robert Wood Johnson Medical School, Rutgers UniversityNJ, USA; ^2^School of Medicine, Ain Shams UniversityCairo, Egypt; ^3^School of Social Sciences and Psychology and Marcs Institute for Brain and Behaviour, University of Western SydneySydney, NSW, Australia

**Keywords:** Parkinson’s disease, MAO inhibitors, cognition, working memory, depression, anxiety, quality of life, learning

## Abstract

**Background**: Prior studies report that monoamine oxidases inhibitors (MAO-I) when used as an adjunct to levodopa ameliorate motor symptoms in Parkinson’s disease (PD), but this was not tested in relation to cognitive or psychiatric measures.

**Objective**: Here, we tested the effects of MAO-I as an adjunct to levodopa, in comparison to levodopa or dopamine (DA) agonists alone, on various cognitive, affective and quality of life measures.

**Methods**: We studied three groups of subjects: healthy controls, PD patients on combined levodopa and MAO-I, and PD patients on levodopa or DA agonists only.

**Results**: We found that compared to monotherapy, combined MAO-I and levodopa seemed to improve cognition, including probabilistic learning, working memory and executive functions. There were no differences between the different medication regimes on deterministic learning, attention or memory recall. It was also found that MAO-I as an adjunct to levodopa improves affective measures such as depression, apathy, anxiety and quality of life. Interestingly, this enhancing effect of combined levodopa and MAO-I was more pronounced in PD patients with severe akinesia, compared to patients with severe tremor.

**Conclusion**: Our data are in agreement with (a) the Continuous Dopaminergic Stimulation (CDS) theory which states that continuous stimulation of the basal ganglia enhances motor, psychiatric and cognitive functions in PD patients; and/or (b) findings that MAO-I increase the bioavailability of monoamines that have beneficial effects on motor and behavioral dysfunction in PD.

## Introduction

### Pharmacological therapy for Parkinson’s disease

Parkinson’s disease (PD) is a progressive and neurodegenerative movement disorder associated with a substantial loss of dopamine (DA) neurons in the substantia nigra (Kish et al., 1988), which are essential for regulating the function of the striatum (a major input structure of the basal ganglia), and the control of voluntary movement.

Standard pharmacological medications for PD include levodopa, various kinds of DA agonists, and Monoamine Oxidases Inhibitors (MAO-I), among others. These medications are prescribed as either monotherapy (taken alone) or polytherapy (using more than one medication in combination) (Rinne, 1987). Levodopa is a DA precursor, taken up by DA cells and converted into DA (Trugman et al., 1991; Muriel et al., 2002; Grace, 2008). MAO- I are a class of chemicals that inhibit the activity of monoamine oxidase enzymes, thus preventing the breakdown of monoamine neurotransmitters, including DA (O’Carroll et al., 1983; Dewey, 2004; Robottom, 2011). MAO-B inhibitors are often administered in the earlier stages of PD, including selegiline and rasagiline. Both these drugs are used either as monotherapy or in combination with levodopa (Caslake et al., 2009; Riederer and Laux, 2011; Fabbrini et al., 2012). While the beneficial effects of these drugs on the motor symptoms of PD are well established, their effects on cognitive and affective symptoms are not as thoroughly investigated.

### Effects of MAO-B Inhibitors on motor, cognitive, and affective processes in Parkinson’s disease

As monotherapy, MAO-B inhibitors may be more effective at the early stages of PD and can delay the need for levodopa. When taken in combination with levodopa (especially as it is usually done in advanced stages of PD), they are known to prolong the effectiveness of levodopa, reduce the amount of levodopa required to control the symptoms and reduce motor fluctuations (Henchcliffe et al., 2005; Riederer and Laux, 2011). While the effectiveness of selegiline in controlling the motor symptoms of PD has been reported as early as 1975 with the first clinical trial by Birkmayer et al. (1975), the effect of rasagiline on motor processes was recently confirmed by several large multicenters as well as smaller clinical trials (Parkinson Study Group, 2002; Olanow et al., 2009). Notable among these studies which used rasagiline as a monotherapy are the ADAGIO and TEMPO studies. The TEMPO study showed the effectiveness of rasagiline on motor processes (as measured by the UPDRS scores), over a 26-week double blind placebo controlled clinical trial on 404 patients with early PD (Parkinson Study Group, 2002). Further, the ADAGIO study noted that 1 mg of MAO-B-I might have a disease modifying effect as observed in activities of daily living scale and the rate of change in the UPDRS scale (Olanow et al., 2009). Importantly, a recent meta-analysis study found that MAO-B inhibitors as an adjunct to levodopa is superior to levodopa alone at reducing PD symptoms in PD patients (Talati et al., 2009).

Research on the effect of MAO Inhibitors on cognitive, behavioral as well as emotional functions in PD has been limited. Most of these studies focus on the effectiveness of the drugs to reduce neuropsychiatric symptoms (Hindmarch et al., 1992; Parkinson Study Group, 1993; Elmer et al., 2006). MAO-B inhibitors such as moclobemide, tranylcypromine, seligiline, and rasagiline (taken alone or in combination with other antidepressants) have been found to be useful in treating depression and anxiety in PD (Steur and Ballering, 1997; Fahn and Chouinard, 1998; Nayak and Henchcliffe, 2008; Korchounov et al., 2012).

Due to its amphetamine-like derivatives, MAO-B Inhibitors have been suggested to improve cognitive performance in cognitively-impaired rats and other subjects (Yasar et al., 1996). Studies conducted on mouse models with derivatives of MAO Inhibitors have shown neuroprotective effects that additionally lend support to the hypothesis of the potential of MAO inhibitors in affecting cognitive, behavioral and emotional measures in patients with PD (Youdim, 2006, 2013; Kupershmidt et al., 2012). Further, Nickel et al. (1990) have shown that in rats, l-deprenyl and l-amphetamine (metabolites of MAOC-I, such as selegiline) increase EEG theta rhythms, indicating that the drug has facilitory effects on learning and memory.

The purpose of this present study is to examine the combined effects of MAO-B inhibitors and levodopa vs. monotherapy involving either DA agonists or levodopa on the motor, psychiatric, and cognitive processes in patients with PD. For this we designed an exploratory study where we recruited three groups of subjects: healthy controls, PD patients on MAO-I and levodopa, and PD patients on levodopa or DA agonists only. All groups were tested on various cognitive, neuropsychological, and affective processes, as described below.

## Methods

### Subjects

The study was approved by the local medical ethics committee, and informed consent was obtained from all subjects, in compliance with research standards for human research at Ain Shams and Cairo Universities. All subjects were recruited from the clinics associated with the Institute of Psychiatry, Ain Shams University as well as Cairo University. The patients diagnosed by a neurologist as having idiopathic PD according to UK Brain Bank diagnostic criteria for PD and they were in three different treatment regimes such as: (1) a monotherapy of levodopa; (2) monotherapy of DA agonists; and (3) combined therapy of levodopa and MAO- I (selegiline or rasagiline). PD patients were assigned different medications to manage their symptoms (tremor, akinesia, gait disturbance, and postural instability). Most patients were initially prescribed levodopa. If levodopa did not manage the symptoms (e.g., based on patients’ distress or caregiver’s observations), they were given MAO-I in addition to it. Some patients were taken off levodopa and assigned DA agonists to manage their symptoms.

We tested healthy controls, PD patients on levodopa and MAO-I (selegiline or rasagiline), and PD patients on monotherapy (levodopa or DA agonists only), using a between-subject design (see Table 1). Among PD patients on monotherapy, 35 subjects were on levodopa while 4 subjects were on DA agonists only (Pramipexole and requip). Among 37 PD patients on levodopa and MAO-I, 19 patients were on selegiline and levodopa and were 18 patients on rasagiline and levodopa. In neurological and clinical practice, the dosage of each selegiline and rasagiline is different. Most of our patients were on daily dose of selegiline of 10 mg (except one patient was on 20 mg daily dose); other patients were on daily dose of either 0.5 or 1 mg of rasagiline. As in our prior studies (Moustafa et al., 2008a,b; Piray et al., 2014), most of our healthy control subjects were spouses of patients, who tended to be fairly well matched demographically. Other healthy control subjects were recruited from the community. The total testing time took approximately 105–120 min for healthy controls, and 115–135 min for PD patients (over two sessions of testing).

**Table 1:** **Subject demographic, clinical and data for healthy controls, PD patients on MAO-I and levodopa, and PD patients on monotherapy (levodopa or dopamine agonists only)**.

	**PD Patients on MAO-I and Ldopa**	**PD Patients on Ldopa or DA agonists only**	**Healthy Controls**	***P*-value**
*N*	37	39	43	
Age	65.2 (4.6)	67.3 (4.3)	66.9 (5.2)	0.304
Sex (M/F)	26/11	27/12	29/14	0.48
PD (akinetic-rigid/Tremor)	21/16	22/17	–	0.734
Education (years)	13.1 (2.3)	12.9 (2.2)	13.2 (3.1)	0.21
NART-predicted IQ	109.5 (12.7)	114.5 (13.1)	116.5 (9.7)	0.53
MMSE	27.4 (1.1)	27.7 (1.4)	28.2 (1.9)	0.420
NAART	34.2 (11.8)	34.8 (11.4)	36.9 (7.3)	0.312
H and Y	2.61 (0.4)	2.53 (0.5)	–	0.601
UPDRS	18.9 (5.9)	23.8 (5.2)	–	0.094
FOGQ	2.6 (0.6)	3.1 (0.7)	–	0.09
Disease duration	8.61 (4.1)	8.46 (3.8)	–	0.732

*Values here represent Mean (S.D). Abbreviations: H and Y, Hoehn and Yahr staging of PD; NAART, North American Adult Reading Test; MMSE, Mini-Mental State Examination; FOGQ, Freezing of Gait Questionnaire; UPDRS, Unified Parkinson’s Disease Rating Scale*.

Each patient’s disease severity was measured using the Hoehn and Yahr stages (Hoehn and Yahr, 1967) and the Unified Parkinson’s Disease Rating Scale (UPDRS).The severity of freezing of gait episodes was measured using the Freezing of Gait Questionnaire (FOGQ; Giladi et al., 2000) and The National Adult Reading Test was used to measure the premorbid intellectual functioning (predicted intelligence quotient (IQ); Bright et al., 2002).

All subjects were screened for intact general cognitive function and absence of dementia with the Mini-Mental Status Exam (MMSE; Folstein et al., 1975) and were required to obtain a score of at least 26 to be considered for the study. Patients who were on combined levodopa and DA agonist therapy, cholinergic, or serotonergic medications were excluded. Altogether, nine subjects were excluded based on these criteria. In addition, two subjects did not learn one of the learning tasks, so their data was not included in the statistical analysis. Subjects who were on multiple levodopa and DA agonists were also excluded from further testing. The final study sample consisted of 43 healthy controls, 37 PD patients on MAO-I and levodopa, and 39 PD patients on monotherapy, which is either levodopa or DA agonists only (see Table 1). Levodopa equivalent daily dose (LEDD) was calculated as in prior studies (Hobson et al., 2005; Ecker et al., 2009; Weintraub et al., 2010; Moustafa et al., 2013).

The motor symptoms of the selected patients varied. Therefore to avoid this factor confounding the results, we categorized the subjects into tremor-predominant and akinetic-rigid subtypes, using their scores on the UPDRS and included the motor symptoms as an independent variable in the analysis. Specifically, a ratio was computed based on the UPDRS part III tremor score (average for items 20 and 21) and the mean UPDRS akinetic/rigid score (average for items 22–27 and 31). A ratio of >1.0 was considered tremor-dominant, <0.80 akinetic-rigid and 0.80–1.0 mixed. Subjects with mixed rigidity-akinesia and tremor (for similar methods used to subtype patients, see Jankovic et al., 1990; Vakil and Herishanu-Naaman, 1998; Poletti et al., 2012) were excluded from the study.

One-way ANOVA was used to compare demographic and neuropsychological measures between the PD patients on MAO-I and levodopa, PD patients on monotherapy, and healthy control subjects. Chi square statistic was used to compare the gender ratio and the PD type (akinesia vs. tremor-dominant), between the medicated PD patients, unmedicated PD patients, and healthy controls groups (Table 1). There was no significant difference between the groups on any of the demographic or motor measures. Although statistically nonsignificant, patients on MAO-I and levodopa had lesser motor dysfunction (as measured by UPDRS) and gait abnormalities (as measured by FOGQ) than the patients who was on a monotherapy of levodopa (Table 1).

### Behavioral tasks and scales

All subjects performed two learning tasks in a counterbalanced order. All tasks were administered on a PC laptop computer.

#### Probabilistic learning task

Subjects were administered a computer-based probabilistic learning task. On each trial, subjects viewed one of six stimuli, and were instructed to make a right or left button-press (e.g., response X or Y). The feedback was probabilistic. So, a correct response results in either gaining points or no feedback with 20 and 80% probability, respectively. An incorrect response results in gaining points or no feedback with 80 and 20% probability, respectively. The correct response (X or Y) varies across stimuli, and the task had 120 trials.

#### Deterministic learning task

The deterministic learning task was similar to the probabilistic learning task, except that the stimuli were different and the stimulus-feedback relationship was deterministic (i.e., stimuli were 100% predictive of a feedback).

#### Neuropsychological assessment

We used the following tests to measure neuropsychological functions such as verbal and visual attention, learning and memory, working memory as well as executive functions.

##### Trail making test- A and B (Reitan and Wolfson, 1985)

This test which consists of a set of numbered and lettered dots which a subject must connect as fast as possible in a serial order. The test consists of two parts, with Part B requiring more cognitive flexibility than Part A. The test provides information about visual attention, scanning, speed of processing and mental flexibility, a component of executive functioning.

##### Digit span test (D. Wechsler, 1958)

This test taken from the Wechsler Adult Intelligence Scale (Digit Span forward and backward) assesses verbal attention and working memory. The subject has to recall immediately a set of numbers presented in either the order in which it was presented or in a backward order. In this study, both forward and backward tests were administered.

##### Controlled oral word association test (Troyer et al., 1997)

This test which assesses verbal fluency requires a subject to generate words starting with the letters F, A and S. In this study the patients were asked to generate the names of animals starting with the above letters.

##### Logical memory test (Wechsler, 1987)

This subtest from the Wechsler Memory Scale–III, assesses verbal memory through immediate recall, delayed recall and recognition tasks following the examiner reading aloud a passage containing a short story to the subject.

##### California verbal learning test (CVLT) (Delis et al., 1987)

This test of verbal learning and memory involves the oral presentation of a 16 word list and immediate recall of the same over five trials. Free recall of the words was assessed again after a delay of approximately 20 min. Afterwards, cued recall is tested by offering the names of the four semantic categories to guide memory retrieval, and yes–no recognition is assessed by embedding the 16 targets among 28 distractors.

##### n-back

The *n*-back task tests the effects of working memory load on performance (Cohen et al., 1997; Perlstein et al., 2003; Owen et al., 2005). In this task, a sequence of letters is presented to the subjects, one at a time. Here, working memory load was either two or three items, that is, subjects had to evaluate the similarity of each item to the one presented *n*-items previously (*n* = 2 or 3). In the two- and three-back conditions, a target was any letter that was identical to the one presented two or three trials preceding it, respectively. Stimulus encoding and response demands were constant across conditions; only requirements to maintain and update increasingly greater amounts of information at higher loads differed. Pseudorandom sequences of single consonants were presented, and subjects responded to each stimulus, pressing one button to targets and another to no targets. Most subjects did not learn the three-back condition, so we do not analyze it any further in the study here, and focus on group differences and medication effects on the two-back task.

##### Stroop color word test (Stroop, 1935)

This test consists of a white sheet of paper with blocks of colors printed on it in a matrix of rows and columns. The subject in the first trial has to read out the names of the colors in an order across rows or columns. In the second trial, the subject is shown a paper with the names of colors written in incongruous colors, again arranged in a matrix. The subject has to suppress reading the print, but name the color in which it is printed, again in the same serial order as before. Each trials are timed separately and scores computed to assess the color-word interference to study the executive functions such as inhibition and cognitive flexibility.

##### Wisconsin card sorting test (WCST) (Heaton et al., 1993)

This test is used to assess the executive functions- set shifting and category formation. The test consists of cards with geometric patterns which the subject has to match based on a principal he/she thinks is right. On receiving a feedback of right or wrong across several trials, a subject learns to match the cards. The category for matching is then shifted to another category at a certain stage in the test without informing the subject. Number and pattern of errors and correct responses reveals learning about category formation and set shifting abilities.

##### Frontal assessment battery (FAB) (Dubois et al., 2000)

The FAB consists of six subtests assessing different functions related to the frontal lobes such as: (1) conceptualization and abstract reasoning (similarities test); (2) mental flexibility (verbal fluency test); (3) motor programming and executive control of action (Luria motor sequences); (4) resistance to interference (conflicting instructions); (5) inhibitory control (go–no go test); and (6) environmental autonomy (prehension behavior). The FAB has shown a good validity (correlation of *ρ* = 0.82 with the Mattis Dementia Rating Scale) and interrater reliability (*κ* = 0.87).

#### Affective and quality of life measures

We should not say additionally here as in the study design we have already said we are looking into the effects/association of different treatment on/with affective measures Beck Depression Inventory was used to assess depressive symptoms (Beck et al., 1987), Beck Anxiety Inventory (Beck et al., 1988) to assess anxiety, Apathy evaluation scale (Marin et al., 1991) to assess apathy, and the PDQ-39 (Jenkinson et al., 1995) questionnaire to assess quality of life.

### Statistical analysis

For all analyses, we used SPSS as well as SAS v8.0 PROC MIXED. One-way ANOVA was used to compare demographic and neuropsychological measures between the PD patients on MAO-I and levodopa, PD patients on monotherapy, and healthy control subjects. Chi square statistic was used to compare the gender ratio and the PD motor subttype (akinesia vs. tremor-dominant), between the medicated PD patients, unmedicated PD patients, and healthy controls groups.

Between-subject differences were examined using unstructured covariance matrices which do not make any strong assumptions about the variance and correlation of the data unlike structured covariances. Where indicated, we tested for specific planned contrasts. In these contrasts, the number of degrees of freedom reflects the entire sample, and not just the subjects involved in the particular contrast, because the mixed procedure analyses between-subject effects, and controls for other variables of interest that apply across all subjects. This procedure uses all of the data to provide a more stable estimate of the error term. Finally, we conducted interaction analysis of medication regime (MAO-I and levodopa vs. monotherapy) and subtype of PD patients (akinesia- vs. tremor-dominant) with all neuropsychological and psychiatric measures.

## Results

Here, we first present results on the association of medication types (multiple vs. monotherapy) on cognition. We then discuss their differential effects on affective and quality of life measures. Finally, we discuss the association of tremor vs. akinesia severity on the same measures.

### Effects of MAO-I as an adjunct to levodopa on cognition

**Figure 1:**
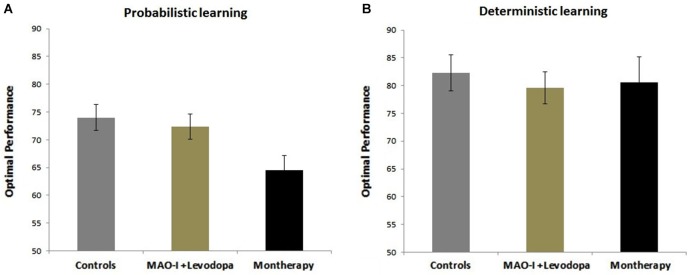
**Effects of combined MAO-B Inhibitors vs. monotherapy on probabilistic and deterministic learning tasks. (A)** Probabilistic learning task. Data show that healthy subjects and PD patients on combined MAO-I and levodopa show better performance than PD patients on monotherapy. **(B)** Deterministic learning task. There was no significant difference among PD and the healthy control groups in the deterministic learning task.

Results show that PD patients on monotherapy were more impaired than healthy subjects and PD patients on combined MAO-I and levodopa in the probabilistic learning task (all *p*’s < 0.01, Figure 1A). There was no significant difference among the healthy controls and PD groups in the deterministic learning task (*p* > 0.1, Figure 1B).

In the case of patients who had MAO-I as an adjunct to levodopa, their neuropsychological measures were better than patients on monotherapy (Table 2). A significant difference was noted on *n*-back task (*p* < 0.01), Backward digit span (*p* = 0.04), Trail Making B (*p* = 0.031), FAB (*p* = 0.031), and WCST-64 (*p* = 0.04). There was no statistically significant difference among the healthy controls and PD groups on other neuropsychological measures (see Table 2).

**Table 2:** **Effects of combined MAO-B Inhibitors vs. monotherapy on cognition**.

	**PD Patients on MAO Inhibitors and Ldopa**	**PD Patients on Ldopa or DA agonists only**	**Healthy Controls**	***P*-value**
Forward digit span	7.1 (1.7)	6.9 (1.4)	6.8 (1.9)	0.37
Backward digit span	7.9 (1.6)	6.12 (1.7)	8.2 (1.8)	0.04
Trail Making A	57.9 (6.3)	75.4 (7.0)	55.8 (6.9)	0.21
Trail Making B	104.1 (18.1)	142.3(18.1)	94.4 (14.3)	0.031
Logical Memory, Delayed Recall	19.7 (7.2)	19.2 (8.1)	18.9 (6.8)	0.54
Logical Memory, Encoding	34.7 (10.9)	35.2 (11.2)	34.1 (12.1)	0.623
Verbal Fluency	18.7 (4.7)	19.6 (4.1)	20.3 (4.2)	0.58
Stroop Errors	5.31 (3.3)	5.1 (2.9)	4.9 (3.1)	0.41
FAB	13.69 (1.4)	10.6 (1.4)	14.1(1.1)	0.031
CVLT-short delay free	7.0 (1.3)	6.9 (1.7)	7.2 (1.3)	0.21
CVLT-long delay free	6.9 (1.2)	6.81 (1.3)	6.9 (1.5)	0.29
CVLT-long delay cued	7.1 (1.0)	7.01 (1.4)	7.1 (1.3)	0.28
CVLT-delayed recognition	8.2 (0.8)	7.9 (0.9)	7.89 (1.3)	0.48
WCST-64	42.8 (10.1)	35.1 (9.3)	44.8 (12.2)	0.04
*n*-back	80.3 (3.35)	74.2 (3.54)	86.2(3.71)	0.007

*Abbreviations: FAB = Frontal Assessment Battery; NART Predicted IQ = National Adult Reading Test predicted intelligence; CVLT = California Verbal Learning Test; WCST-64 = Wisconsin Card Sorting Test-64 cards version*.

### Association of MAO-I as an adjunct to levodopa on affective and quality of life measures

As in the case of neuropsychological measures, patients who had MAO-I as an adjunct to levodopa had better scores on motivational processes than patients on monotherapy (Table 3). Specifically, we found that compared to PD patients on monotherapy, MAO-I as adjunct to levodopa show more beneficial effects on mental health on the following measures: apathy (*p* = 0.032), BDI (*p* = 0.031) and quality of life, as measured using the PD-39 scale (*p* = 0.041). Although statistically nonsignificant, unlike monotherapy, MAO-I as adjunct to levodopa seem to better ameliorate anxiety (*p* = 0.09).

**Table 3:** **Effects of combined MAO-B Inhibitors and levodopa vs. monotherapy on affective and quality of life measures**.

	**PD Patients on MAO-B Inhibitors and Ldopa**	**PD Patients on Ldopa or DA agonists only**	**Healthy Controls**	***P*-value**
Apathy (Apathy evaluation scale)	32.6 (6.2)	39.8 (5.1)	33.4 (5.7)	0.032
Depression (BDI)	6.2 (1.2)	8.9 (1.1)	6.5 (1.9)	0.031
Anxiety (BAI)	14.3 (3.0)	17.2 (4.3)	13.3 (3.1)	0.09
Quality of life (PDQ-39)	29.8 (2.7)	25.3 (2.4)	–	0.041

*Abbreviations: BDI, Beck Depression Inventory; BAI, Beck Anxiety Inventory*.

We further separated the levodopa and MAO-I group into two groups (patients on rasagiline vs. others on selegiline), and tested their performance on all questionnaires and cognitive tests. There were no significant differences among the two groups in any of the measures, except that in the rasagiline and levodopa group, UPDRS scores were slightly but not significantly lower than in patients in the selegiline and levodopa group (*p* = 0.083).

### Interaction of medication regime with motor subtype

On conducting an interaction analysis of medication regime (MAO-I and levodopa vs. monotherapy) and subtype of PD patients (akinesia- vs. tremor-dominant), the ameliorating effects of MAO-I were more pronounced in PD patients with severe akinesia than PD patients with predominant tremor. A significant interaction was found between medication regime and subtype of PD patients on measures of Trail-Making B (*p* < 0.02), FAB (*p* < 0.01), WCST (*p* < 0.04), BDI (*p* < 0.02), and apathy (*p* < 0.009). All other interaction effects (with neuropsychological and psychiatric measures) were not significant (all *p*’s > 0.15).

## Discussion

Our results show MAO-B inhibitors as an adjunct to levodopa provide a more enhancing effect than the use of monotherapy (levodopa or DA agonists alone) on most cognitive, affective and quality of life measures used in our study. Below, we discuss the effects of MAO-B inhibitors as an adjunct to levodopa on neuropsychiatric and cognitive measures.

### Effects of MAO-B inhibitors on cognitive measures

As mentioned above, there are very few studies that have investigated the effects of MAO-B inhibitors (taken alone or as an adjunct to levodopa) on neuropsychological and cognitive measures. For example, rasagiline has been used to treat mild cognitive impairment in PD (Goldman and Holden, 2014). A small study involving seven patients on selegiline showed some improvement in memory and motor speed without progressive memory loss compared to those with dementia (Portin and Rinne, 1983). Subsequently, Hietanen (1991) tested 18 patients with idiopathic PD on selegiline as monotherapy for 8 weeks in a double blind randomized placebo controlled trial, using a battery of neuropsychological tests. The study found some improvement in learning easy word associations, but did not find any significant specific cognitive effect. As part of a multicenter trial of Deprenyl and Tocopherol Antioxidative Therapy of Parkinsonism (DATATOP), 800 patients with early untreated PD were administered tests that measured memory, visuospatial, and frontal lobe functions (Kieburtz et al., 1994). The study did not find any significant effect of either deprenyl (selegiline) or tocopherol on cognitive test performance (Kieburtz et al., 1994). Further, in an 8-week study, it was found that selegiline did not have an effect on executive function using the Wisconsin Card Sorting Test (Dalrymple-Alford et al., 1995). Recently, Hanagasi et al. (2011) found that compared to placebo, the MAO-B inhibitor rasagiline has a better effect on attention and executive function in nondemented PD patients. In this double blind placebo controlled multicenter trial of 48 non-demented patients with PD and cognitive impairment, significant improvement was noticed in the rasagiline group on scores of digit span backward, verbal fluency, semantic fluency, Stroop, and attentional measures (Hanagasi et al., 2011).

Our results extend these findings by showing that MAO-B inhibitors as an adjunct to levodopa have a better effect on cognitive function (including probabilistic learning, forward and backward digit span, *n*-back, WCST-64, FAB, Trail Making B) in PD patients than levodopa or DA agonists monotherapy. Unlike the Hanagasi et al. study, the present study employed computerized cognitive tasks to assess learning in PD patients. Our data show that patients who had MAO-B inhibitors as adjunct to levodopa had better probabilistic but not deterministic learning. Our results suggest that learning impairment is exacerbated when the learning task involves uncertain feedback and response conflict, as in the probabilistic, but not deterministic, learning task. This interpretation is also in agreement with prior research showing that PD patients show impairments in conflict-based decision making tasks (Farooqui et al., 2011; Vandenbossche et al., 2012). This also points to the finding that probabilistic learning has a better potential to reveal differences in drug effects than deterministic learning tasks.

### Effects of MAO-B inhibitors on affective and quality of life measures

MAO inhibitors have been generally used as traditional antidepressant drugs (Johnson et al., 2010). For example, many studies show that selegiline transdermal system help reduce depressive symptoms in various patient populations (Bodkin and Amsterdam, 2002; Amsterdam and Bodkin, 2006; Feiger et al., 2006). In our study, we found that PD patients who were on MAO-I and levodopa have lower BDI scores than patients on levodopa or DA agonists alone, suggesting a beneficial effect of MAO inhibitors on depression. Although not statistically significant, we found that PD patients on MAO-I and levodopa have lower scores on anxiety measure (as used by the Beck anxiety Inventory questionnaire), when compared to patients on monotherapy.

Interestingly, we also found that PD patients who were on MAO-I and levodopa have lower apathy and higher quality of life scores than patients on levodopa or DA agonists alone, suggesting a beneficial effect of MAO inhibitors on these measures.

### Interaction between motor subtype in PD and medication regime

We also found an interaction effect in the motor subtype, medication and cognitive functions. Better cognitive functions were seen more in patients with severe akinesia, in comparison to patients with severe tremor. Our results extend prior studies showing relationships between motor and cognitive measures in PD (Riggeal et al., 2007; Colman et al., 2009; Wylie et al., 2012; Smulders et al., 2013), and further show that the effects of MAO-I on neuropsychological, affective, and quality of life measures in PD depends on the subtypes of PD.

Our results are also in line with prior studies showing that PD patients with tremor are usually less cognitively impaired than PD patients with akinesia or gait dysfunction (Vakil and Herishanu-Naaman, 1998; Burn et al., 2006; Lyros et al., 2008; Oh et al., 2009; Domellof et al., 2011). In addition, prior studies have suggested that akinesia in PD patients is related to basal ganglia dysfunction (Kassubek et al., 2002; Probst-Cousin et al., 2003; Weinberger et al., 2009; Zaidel et al., 2009; Mure et al., 2011). It is possible that in PD patients with severe akinesia, the combined therapy of MAO inhibitors and levodopa has a more ameliorative effect on basal ganglia dysfunction than levodopa alone. We hypothesize that this effect can perhaps be due to findings that tremor in PD is related to damage to brain areas such as the cerebellum, while akinesia has been consistently seen to be related to basal ganglia dysfunction (Mure et al., 2011). Further, it is possible that MAO-B inhibitors ameliorate the function of the basal ganglia and DA and thus ameliorate impairment in PD patients with akinesia that is patients with more cognitive damage get better effects from treatment.

### Continuous dopaminergic stimulation theory and bioavailability of monoamines

The beneficial effects of MAO-I can be due to the Continuous Dopaminergic Stimulation (CDS) of the basal ganglia and/or an increase in the bioavailability of monoamines. The CDS theory posits that sufficient dopaminergic stimulation of the basal ganglia (and particularly the striatum) reduces the occurrence of motor complications (such as wearing-off phenomenon) and dyskinesia (Nyholm, 2007; Silverdale, 2007). A multitude of studies have shown that these motor complications are associated with the administration of levodopa medications, and that combined therapies (levodopa with DA agonists, COMT inhibitors, or MAO inhibitors) can reduce these motor fluctuations (Jankovic and Stacy, 2007; Stocchi et al., 2008).

Alternatively, many studies have shown that MAO-I can increase the levels of many monoamines including DA, serotonin, and norepinephrine (Riederer and Laux, 2011). Most of these monoamines are known to impact cognitive and psychiatric measures in various patient populations, and thus the beneficial effects of MAO-I on our patients could be due to an increase of monoamine levels in the brain (Hamon and Blier, 2013).

Our results extend these findings, and show that the beneficiary effects of levodopa and MAO-I can also be observed in cognitive, affective and quality of life measures. It is possible that MAO-I and levodopa enhance DA and other monoamine neurotransmission and thus provide a continuous stimulation of the basal ganglia, which in turn, ameliorate neuropsychological, affective and quality of life dysfunction in PD patients. None of our patients were on COMT inhibitors, but future studies should evaluate whether COMT inhibitors (taken alone or in combination with levodopa) also ameliorate neuropsychiatric and neuropsychological function in PD patients, in comparison to monotherapy.

### Limitations

Our study is not without limitations. Our samples included a small number of subjects to compare PD patients on levodopa to patients on DA agonists alone. There are very few number of PD patients on DA agonists (either alone or in combination with other medications). A future study recruiting patients on DA agonists and/or MAO-B inhibitors can help understand its effects on motor, psychiatric, and cognitive processes in comparison to levodopa and/or MAO-B inhibitors. Similarly, we did not have a large sample of PD patients to compare differential effects of selegiline vs. rasagiline on neurocognitive and neuropsychiatric measures. Further, a longitudinal study is needed to confirm findings that the administration of MAO-I and levodopa better ameliorate and cognitive abnormalities than the administration of levodopa alone.

Overall, our results show that combination therapy of MAO inhibitors and levodopa are associated with better neuropsychological, cognitive, and affective function in PD patients than levodopa or DA agonists alone. To our knowledge, this is the first study to investigate the effects of MAO inhibitors on cognitive as well as classical neuropsychological tests in PD patients using computerized learning tests.

## Conflict of interest statement

The authors declare that the research was conducted in the absence of any commercial or financial relationships that could be construed as a potential conflict of interest.

## References

[B1] Amsterdam J. D. Bodkin J. A. (2006). Selegiline transdermal system in the prevention of relapse of major depressive disorder: a 52-week, double-blind, placebo-substitution, parallel-group clinical trial. J. Clin. Psychopharmacol. 26, 579–586 10.1097/01.jcp.0000239794.37073.70 17110814

[B87] Beck D. C. Carlson G. A. Russell A. T. Brownfield F. E. (1987). Use of depression rating instruments in developmentally and educationally delayed adolescents. J. Am. Acad. Child Adolesc. Psychiatry 26, 97–100 10.1097/00004583-198701000-00019 3584006

[B2] Beck A. T. Epstein N. Brown G. Steer R. A. (1988). An inventory for measuring clinical anxiety: psychometric properties. J. Consult. Clin. Psychol. 56, 893–897 10.1037/0022-006x.56.6.893 3204199

[B3] Birkmayer W. Riederer P. Youdim M. B. Linauer W. (1975). The potentiation of the anti akinetic effect after L-dopa treatment by an inhibitor of MAO-B, deprenil. J. Neural Transm. 36, 303–326 10.1007/bf01253131 1172524

[B4] Bodkin J. A. Amsterdam J. D. (2002). Transdermal selegiline in major depression: a double-blind, placebo-controlled, parallel-group study in outpatients. Am. J. Psychiatry 159, 1869–1875 10.1176/appi.ajp.159.11.1869 12411221

[B5] Bright P. Jaldow E. Kopelman M. D. (2002). The national adult reading test as a measure of premorbid intelligence: a comparison with estimates derived from demographic variables. J. Int. Neuropsychol. Soc. 8, 847–854 10.1017/s1355617702860131 12240749

[B6] Burn D. J. Rowan E. N. Allan L. M. Molloy S. O’Brien J. T. McKeith I. G. (2006). Motor subtype and cognitive decline in Parkinson’s disease, Parkinson’s disease with dementia and dementia with Lewy bodies. J. Neurol. Neurosurg. Psychiatry 77, 585–589 10.1136/jnnp.2005.081711 16614017 PMC2117449

[B7] Caslake R. Macleod A. Ives N. Stowe R. Counsell C. (2009). Monoamine oxidase B inhibitors versus other dopaminergic agents in early Parkinson’s disease. Cochrane Database Syst. Rev. 4:CD006661 10.1002/14651858.CD006661 19821381 PMC13500180

[B8] Cohen J. D. Perlstein W. M. Braver T. S. Nystrom L. E. Noll D. C. Jonides J. (1997). Temporal dynamics of brain activation during a working memory task. Nature 386, 604–608 10.1038/386604a0 9121583

[B9] Colman K. S. Koerts J. van Beilen M. Leenders K. L. Post W. J. Bastiaanse R. (2009). The impact of executive functions on verb production in patients with Parkinson’s disease. Cortex 45, 930–942 10.1016/j.cortex.2008.12.010 19303593

[B10] Dalrymple-Alford J. C. Jamieson C. F. Donaldson I. M. (1995). Effects of selegiline (deprenyl) on cognition in early Parkinson’s disease. Clin. Neuropharmacol. 18, 348–359 10.1097/00002826-199508000-00007 8665548

[B11] Delis D. C. Kramer J. H. Kaplan E. Ober B. A. (1987). The California Verbal Learning Test. San Antonio, TX: Psychological Corporation

[B12] Dewey R. B. Jr. (2004). Management of motor complications in Parkinson’s disease. Neurology 62(6 Suppl. 4), S3–S7 10.1212/wnl.62.6_suppl_4.s3 15037664

[B13] Domellof M. E. Elgh E. Forsgren L. (2011). The relation between cognition and motor dysfunction in drug-naive newly diagnosed patients with Parkinson’s disease. Mov. Disord. 26, 2183–2189 10.1002/mds.23814 21661051

[B14] Dubois B. Slachevsky A. Litvan I. Pillon B. (2000). The FAB: a Frontal Assessment Battery at bedside. Neurology 55, 1621–1626 10.1212/wnl.55.11.1621 11113214

[B15] Ecker D. Unrath A. Kassubek J. Sabolek M. (2009). Dopamine agonists and their risk to induce psychotic episodes in Parkinson’s disease: a case-control study. BMC Neurol. 9:23 10.1186/1471-2377-9-23 19515253 PMC2704166

[B16] Elmer L. Schwid S. Eberly S. Goetz C. Fahn S. Kieburtz K. (2006). Rasagiline-associated motor improvement in PD occurs without worsening of cognitive and behavioral symptoms. J. Neurol. Sci. 248, 78–83 10.1016/j.jns.2006.05.014 16828804

[B17] Fabbrini G. Abbruzzese G. Marconi S. Zappia M. (2012). Selegiline: a reappraisal of its role in Parkinson disease. Clin. Neuropharmacol. 35, 134–140 10.1097/WNF.0b013e318255838b 22592509

[B18] Fahn S. Chouinard S. (1998). Experience with tranylcypromine in early Parkinson’s disease. J. Neural Transm. Suppl. 52, 49–61 10.1007/978-3-7091-6499-0_6 9564607

[B19] Farooqui A. A. Bhutani N. Kulashekhar S. Behari M. Goel V. Murthy A. (2011). Impaired conflict monitoring in Parkinson’s disease patients during an oculomotor redirect task. Exp. Brain Res. 208, 1–10 10.1007/s00221-010-2432-y 21082315

[B86] Folstein M. F. Folstein S. E. McHugh P. R. (1975). “Mini-mental state”. A practical method for grading the cognitive state of patients for the clinician. J. Psychiatr. Res. 12, 189–198 10.1016/0022-3956(75)90026-6 1202204

[B20] Feiger A. D. Rickels K. Rynn M. A. Zimbroff D. L. Robinson D. S. (2006). Selegiline transdermal system for the treatment of major depressive disorder: an 8-week, double-blind, placebo-controlled, flexible-dose titration trial. J. Clin. Psychiatry 67, 1354–1361 10.4088/jcp.v67n0905 17017821

[B21] Giladi N. Shabtai H. Simon E. S. Biran S. Tal J. Korczyn A. D. (2000). Construction of freezing of gait questionnaire for patients with Parkinsonism. Parkinsonism Relat. Disord. 6, 165–170 10.1016/s1353-8020(99)00062-0 10817956

[B22] Goldman J. G. Holden S. (2014). Treatment of psychosis and dementia in Parkinson’s disease. Curr. Treat. Options Neurol. 16:281 10.1007/s11940-013-0281-2 24464490 PMC3994190

[B23] Grace A. A. (2008). Physiology of the normal and dopamine-depleted basal ganglia: insights into levodopa pharmacotherapy. Mov. Disord. 23(Suppl. 3), S560–S569 10.1002/mds.22020 18781673

[B24] Hamon M. Blier P. (2013). Monoamine neurocircuitry in depression and strategies for new treatments. Prog. Neuropsychopharmacol. Biol. Psychiatry 45, 54–63 10.1016/j.pnpbp.2013.04.009 23602950

[B25] Hanagasi H. A. Gurvit H. Unsalan P. Horozoglu H. Tuncer N. Feyzioglu A. (2011). The effects of rasagiline on cognitive deficits in Parkinson’s disease patients without dementia: a randomized, double-blind, placebo-controlled, multicenter study. Mov. Disord. 26, 1851–1858 10.1002/mds.23738 21500280

[B26] Heaton S. K. Chelune G. J. Talley J. L. Kay G. G. Curtiss G. (1993). Wisconsin Card Sorting Test Manual: Revised and Expanded. Odessa, FL: Psychological Assessment Resources

[B27] Henchcliffe C. Schumacher H. C. Burgut F. T. (2005). Recent advances in Parkinson’s disease therapy: use of monoamine oxidase inhibitors. Expert Rev. Neurother. 5, 811–821 10.1586/14737175.5.6.811 16274338

[B28] Hietanen M. H. (1991). Selegiline and cognitive function in Parkinson’s disease. Acta Neurol. Scand. 84, 407–410 10.1111/j.1600-0404.1991.tb04978.x 1776388

[B29] Hindmarch I. Alford C. Barwell F. Kerr J. S. (1992). Measuring the side effects of psychotropics: the behavioural toxicity of antidepressants. J. Psychopharmacol. 6, 198–203 10.1177/026988119200600212 22291351

[B30] Hobson P. Gallacher J. Meara J. (2005). Cross-sectional survey of Parkinson’s disease and parkinsonism in a rural area of the United Kingdom. Mov. Disord. 20, 995–998 10.1002/mds.20489 15852368

[B31] Hoehn M. M. Yahr M. D. (1967). Parkinsonism: onset, progression and mortality. Neurology 17, 427–442 10.1212/WNL.17.5.427 6067254

[B32] Jankovic J. McDermott M. Carter J. Gauthier S. Goetz C. Golbe L. (1990). Variable expression of Parkinson’s disease: a base-line analysis of the DATATOP cohort. The Parkinson study group. Neurology 40, 1529–1534 10.1212/wnl.40.10.1529 2215943

[B33] Jankovic J. Stacy M. (2007). Medical management of levodopa-associated motor complications in patients with Parkinson’s disease. CNS Drugs 21, 677–692 10.2165/00023210-200721080-00005 17630819

[B34] Jenkinson C. Peto V. Fitzpatrick R. Greenhall R. Hyman N. (1995). Self-reported functioning and well-being in patients with Parkinson’s disease: comparison of the short-form health survey (SF-36) and the Parkinson’s Disease Questionnaire (PDQ-39). Age Ageing 24, 505–509 10.1093/ageing/24.6.505 8588541

[B35] Johnson S. Tazik S. Lu D. Johnson C. Youdim M. B. Wang J. (2010). The new inhibitor of monoamine oxidase, M30, has a neuroprotective effect against dexamethasone-induced brain cell apoptosis. Front. Neurosci. 4:180 10.3389/fnins.2010.00180 21103012 PMC2987595

[B36] Kassubek J. Juengling F. D. Hellwig B. Spreer J. Lucking C. H. (2002). Thalamic gray matter changes in unilateral Parkinsonian resting tremor: a voxel-based morphometric analysis of 3-dimensional magnetic resonance imaging. Neurosci. Lett. 323, 29–32 10.1016/s0304-3940(02)00111-8 11911983

[B37] Kieburtz K. McDermott M. Como P. Growdon J. Brady J. Carter J. (1994). The effect of deprenyl and tocopherol on cognitive performance in early untreated Parkinson’s disease. Parkinson study group. Neurology 44, 1756–1759 10.1212/wnl.44.9.1756 7936311

[B38] Kish S. J. Shannak K. Hornykiewicz O. (1988). Uneven pattern of dopamine loss in the striatum of patients with idiopathic Parkinson’s disease. Pathophysiologic and clinical implications. N. Engl. J. Med. 318, 876–880 10.1056/nejm198804073181402 3352672

[B39] Korchounov A. Winter Y. Rossy W. (2012). Combined beneficial effect of rasagiline on motor function and depression in de novo PD. Clin. Neuropharmacol. 35, 121–124 10.1097/WNF.0b013e31823b1da8 22561875

[B40] Kupershmidt L. Amit T. Bar-Am O. Weinreb O. Youdim M. B. (2012). Multi-target, neuroprotective and neurorestorative M30 improves cognitive impairment and reduces Alzheimer’s-like neuropathology and age-related alterations in mice. Mol. Neurobiol. 46, 217–220 10.1007/s12035-012-8304-7 22847630

[B41] Lyros E. Messinis L. Papathanasopoulos P. (2008). Does motor subtype influence neurocognitive performance in Parkinson’s disease without dementia? Eur. J. Neurol. 15, 262–267 10.1111/j.1468-1331.2007.02046.x 18190508

[B42] Marin R. S. Biedrzycki R. C. Firinciogullari S. (1991). Reliability and validity of the apathy evaluation scale. Psychiatry Res. 38, 143–162 10.1016/0165-1781(91)90040-v 1754629

[B43] Moustafa A. A. Bell P. Eissa A. M. Hewedi D. H. (2013). The effects of clinical motor variables and medication dosage on working memory in Parkinson’s disease. Brain Cogn. 82, 137–145 10.1016/j.bandc.2013.04.001 23660434

[B44] Moustafa A. A. Cohen M. X. Sherman S. J. Frank M. J. (2008a). A role for dopamine in temporal decision making and reward maximization in parkinsonism. J. Neurosci. 28, 12294–12304 10.1523/JNEUROSCI.3116-08.2008 19020023 PMC3049941

[B45] Moustafa A. A. Sherman S. J. Frank M. J. (2008b). A dopaminergic basis for working memory, learning and attentional shifting in Parkinsonism. Neuropsychologia 46, 3144–3156 10.1016/j.neuropsychologia.2008.07.011 18687347

[B46] Mure H. Hirano S. Tang C. C. Isaias I. U. Antonini A. Ma Y. (2011). Parkinson’s disease tremor-related metabolic network: characterization, progression and treatment effects. Neuroimage 54, 1244–1253 10.1016/j.neuroimage.2010.09.028 20851193 PMC2997135

[B47] Muriel M. P. Orieux G. Hirsch E. C. (2002). Levodopa but not ropinirole induces an internalization of D1 dopamine receptors in parkinsonian rats. Mov. Disord. 17, 1174–1179 10.1002/mds.10256 12465054

[B48] Nayak L. Henchcliffe C. (2008). Rasagiline in treatment of Parkinson’s disease. Neuropsychiatr. Dis. Treat. 4, 23–32 18728823 PMC2515917

[B49] Nickel B. Schulze G. Szelenyi I. (1990). Effect of enantiomers of deprenyl (selegiline) and amphetamine on physical abuse liability and cortical electrical activity in rats. Neuropharmacology 29, 983–992 10.1016/0028-3908(90)90103-x 2128372

[B50] Nyholm D. (2007). The rationale for continuous dopaminergic stimulation in advanced Parkinson’s disease. Parkinsonism Relat. Disord. 13(Suppl), S13–S17 10.1016/j.parkreldis.2007.06.005 17707679

[B51] O’Carroll A. M. Fowler C. J. Phillips J. P. Tobbia I. Tipton K. F. (1983). The deamination of dopamine by human brain monoamine oxidase. Specificity for the two enzyme forms in seven brain regions. Naunyn Schmiedebergs Arch. Pharmacol. 322, 198–202 10.1007/bf00500765 6408492

[B52] Oh J. Y. Kim Y. S. Choi B. H. Sohn E. H. Lee A. Y. (2009). Relationship between clinical phenotypes and cognitive impairment in Parkinson’s disease (PD). Arch. Gerontol. Geriatr. 49, 351–354 10.1016/j.archger.2008.11.013 19136159

[B53] Olanow C. W. Rascol O. Hauser R. Feigin P. D. Jankovic J. Lang A. (2009). A double-blind, delayed-start trial of rasagiline in Parkinson’s disease. N. Engl. J. Med. 361, 1268–1278 10.1056/NEJMoa0809335 19776408

[B54] Owen A. M. McMillan K. M. Laird A. R. Bullmore E. (2005). N-back working memory paradigm: a meta-analysis of normative functional neuroimaging studies. Hum. Brain Mapp. 25, 46–59 10.1002/hbm.20131 15846822 PMC6871745

[B55] Parkinson Study Group (1993). Effects of tocopherol and deprenyl on the progression of disability in early Parkinson’s disease. The Parkinson study group. N. Engl. J. Med. 328, 176–183 10.1056/NEJM199301213280305 8417384

[B56] Parkinson Study Group (2002). A controlled trial of rasagiline in early Parkinson disease: the TEMPO study. Arch. Neurol. 59, 1937–1943 10.1001/archneur.59.12.1937 12470183

[B57] Perlstein W. M. Dixit N. K. Carter C. S. Noll D. C. Cohen J. D. (2003). Prefrontal cortex dysfunction mediates deficits in working memory and prepotent responding in schizophrenia. Biol. Psychiatry 53, 25–38 10.1016/s0006-3223(02)01675-x 12513942

[B58] Piray P. Zeighami Y. Bahrami F. Eissa A. M. Hewedi D. H. Moustafa A. A. (2014). Impulse control disorders in Parkinson’s disease are associated with dysfunction in stimulus valuation but not action valuation. J. Neurosci. 34, 7814–7824 10.1523/JNEUROSCI.4063-13.2014 24899705 PMC6608260

[B59] Poletti M. Frosini D. Pagni C. Baldacci F. Nicoletti V. Tognoni G. (2012). Mild cognitive impairment and cognitive-motor relationships in newly diagnosed drug-naive patients with Parkinson’s disease. J. Neurol. Neurosurg. Psychiatry 83, 601–606 10.1136/jnnp-2011-301874 22492216

[B60] Portin R. Rinne U. K. (1983). The effect of deprenyl (selegiline) on cognition and emotion in parkinsonian patients undergoing long-term levodopa treatment. Acta Neurol. Scand. Suppl. 95, 135–144 10.1111/j.1600-0404.1983.tb01528.x 6428146

[B61] Probst-Cousin S. Druschky A. Neundorfer B. (2003). Disappearance of resting tremor after “stereotaxic” thalamic stroke. Neurology 61, 1013–1014 10.1212/01.wnl.0000086810.14643.fc 14557586

[B62] Reitan R. M. Wolfson D. (1985). The Halstead-Reitan Neuropsychological Test Battery: Theory and Clinical Interpretation. Tucson, Ariz.: Neuropsychology Press

[B63] Riederer P. Laux G. (2011). MAO-inhibitors in Parkinson’s disease. Exp. Neurobiol. 20, 1–17 10.5607/en.2011.20.1.1 22110357 PMC3213739

[B64] Riggeal B. D. Crucian G. P. Seignourel P. Jacobson C. E. Okun M. S. Rodriguez R. (2007). Cognitive decline tracks motor progression and not disease duration in Parkinson patients. Neuropsychiatr. Dis. Treat. 3, 955–958 19300633 10.2147/ndt.s2237 PMC2656340

[B65] Rinne U. K. (1987). Early combination of bromocriptine and levodopa in the treatment of Parkinson’s disease: a 5-year follow-up. Neurology 37, 826–828 10.1212/wnl.37.5.826 3574685

[B66] Robottom B. J. (2011). Efficacy, safety and patient preference of monoamine oxidase B inhibitors in the treatment of Parkinson’s disease. Patient Prefer. Adherence 5, 57–64 10.2147/PPA.s11182 21423589 PMC3058602

[B67] Silverdale M. (2007). Continuous dopaminergic stimulation in Parkinson’s disease. Prog. Neurol. Psychiatry 11, 24–28 10.1002/pnp.3

[B68] Smulders K. van Nimwegen M. Munneke M. Bloem B. R. Kessels R. P. Esselink R. A. (2013). Involvement of specific executive functions in mobility in Parkinson’s disease. Parkinsonism Relat. Disord. 19, 126–128 10.1016/j.parkreldis.2012.06.010 22771282

[B69] Steur E. N. Ballering L. A. (1997). Moclobemide and selegeline in the treatment of depression in Parkinson’s disease. J. Neurol. Neurosurg. Psychiatry 63:547 10.1136/jnnp.63.4.547 9343144 PMC2169782

[B70] Stocchi F. Tagliati M. Olanow C. W. (2008). Treatment of levodopa-induced motor complications. Mov. Disord. 23(Suppl. 3), S599–S612 10.1002/mds.22052 18781681

[B71] Stroop J. R. (1935). Studies of interference in serial verbal reactions. J. Exp. Psychol. 18, 643–662 10.1037/h0054651

[B72] Talati R. Reinhart K. Baker W. White C. M. Coleman C. I. (2009). Pharmacologic treatment of advanced Parkinson’s disease: a meta-analysis of COMT inhibitors and MAO-B inhibitors. Parkinsonism Relat. Disord. 15, 500–505 10.1016/j.parkreldis.2008.12.007 19167259

[B73] Troyer A. K. Moscovitch M. Winocur G. (1997). Clustering and switching as two components of verbal fluency: evidence from younger and older healthy adults. Neuropsychology 11, 138–146 10.1037//0894-4105.11.1.138 9055277

[B74] Trugman J. M. James C. L. Wooten G. F. (1991). D1/D2 dopamine receptor stimulation by L-dopa. A [14C]-2-deoxyglucose autoradiographic study. Brain 114(Pt. 3), 1429–1440 10.1093/brain/114.3.1429 1829645

[B75] Vakil E. Herishanu-Naaman S. (1998). Declarative and procedural learning in Parkinson’s disease patients having tremor or bradykinesia as the predominant symptom. Cortex 34, 611–620 10.1016/s0010-9452(08)70518-5 9800094

[B76] Vandenbossche J. Deroost N. Soetens E. Zeischka P. Spildooren J. Vercruysse S. (2012). Conflict and freezing of gait in Parkinson’s disease: support for a response control deficit. Neuroscience 206, 144–154 10.1016/j.neuroscience.2011.12.048 22265727

[B77] Wechsler D. (1958). The Measurement and Appraisal of Adult Intelligence. 4th Edn. Baltimore: Williams and Wilkins

[B78] Wechsler D. (1987). Wechsler Memory Scale-Revised. San Antonio, TX: Psychological Corporation

[B79] Weinberger M. Hutchison W. D. Lozano A. M. Hodaie M. Dostrovsky J. O. (2009). Increased gamma oscillatory activity in the subthalamic nucleus during tremor in Parkinson’s disease patients. J. Neurophysiol. 101, 789–802 10.1152/jn.90837.2008 19004998

[B80] Weintraub D. Koester J. Potenza M. N. Siderowf A. D. Stacy M. Voon V. (2010). Impulse control disorders in Parkinson disease: a cross-sectional study of 3090 patients. Arch. Neurol. 67, 589–595 10.1001/archneurol.2010.65 20457959

[B81] Wylie S. A. van den Wildenberg W. Ridderinkhof K. R. Claassen D. O. Wooten G. F. Manning C. A. (2012). Differential susceptibility to motor impulsivity among functional subtypes of Parkinson’s disease. J. Neurol. Neurosurg. Psychiatry 83, 1149–1154 10.1136/jnnp-2012-303056 22917670 PMC3704227

[B82] Yasar S. Goldberg J. P. Goldberg S. R. (1996). Are metabolites of l-deprenyl (selegiline) useful or harmful? Indications from preclinical research. J. Neural Transm. Suppl. 48, 61–73 10.1007/978-3-7091-7494-4_6 8988462

[B83] Youdim M. B. (2006). The path from anti Parkinson drug selegiline and rasagiline to multifunctional neuroprotective anti Alzheimer drugs ladostigil and m30. Curr. Alzheimer Res. 3, 541–550 10.2174/156720506779025288 17168653

[B84] Youdim M. B. (2013). Multi target neuroprotective and neurorestorative anti-Parkinson and anti-Alzheimer drugs ladostigil and m30 derived from rasagiline. Exp. Neurobiol. 22, 1–10 10.5607/en.2013.22.1.1 23585716 PMC3620452

[B85] Zaidel A. Arkadir D. Israel Z. Bergman H. (2009). Akineto-rigid vs. tremor syndromes in Parkinsonism. Curr. Opin. Neurol. 22, 387–393 10.1097/WCO.0b013e32832d9d67 19494773

